# Physician reports of medication use with explicit intention of hastening the end of life in the absence of explicit patient request in general practice in Belgium

**DOI:** 10.1186/1471-2458-10-186

**Published:** 2010-04-09

**Authors:** Koen Meeussen, Lieve Van den Block, Nathalie Bossuyt, Michael Echteld, Johan Bilsen, Luc Deliens

**Affiliations:** 1 End-of-Life Care Research Group, Vrije Universiteit Brussel, Brussels, Belgium; 2 Department of General Practice, Vrije Universiteit Brussel, Brussels, Belgium; 3 Scientific Institute of Public Health, Unit of Epidemiology, Brussels, Belgium; 4 VU University Medical Centre, Department of Public and Occupational Health, EMGO Institute for Health and Care Research, Expertise Centre for Palliative Care, Amsterdam, the Netherlands; 5 Department of Public health, Vrije Universiteit Brussel, Brussels, Belgium

## Abstract

**Background:**

Although the incidence of the use of life-ending drugs without explicit patient request has been estimated in several studies, in-depth empirical research on this controversial practice is nonexistent. Based on face-to-face interviews with the clinicians involved in cases where patients died following such a decision in general practice in Belgium, we investigated the clinical characteristics of the patients, the decision-making process, and the way the practice was conducted.

**Methods:**

Mortality follow-back study in 2005-2006 using the nationwide Sentinel Network of General Practitioners, a surveillance instrument representative of all GPs in Belgium. Standardised face-to-face interviews were conducted with all GPs who reported a non-sudden death in their practice, at home or in a care home, which was preceded by the use of a drug prescribed, supplied or administered by a physican without an explicit patient request.

**Results:**

Of the 2690 deaths registered by the GPs, 17 were eligible to be included in the study. Thirteen interviews were conducted. GPs indicated that at the time of the decision all patients were without prospect of improvement, with persistent and unbearable suffering to a (very) high degree in nine cases. Twelve patients were judged to lack the competence to make decisions. GPs were unaware of their patient's end-of-life wishes in nine cases, but always discussed the practice with other caregivers and/or the patient's relatives. All but one patient received opioids to hasten death. All GPs believed that end-of-life quality had been "improved considerably".

**Conclusions:**

The practice of using life-ending drugs without explicit patient request in general practice in Belgium mainly involves non-competent patients experiencing persistent and unbearable suffering whose end-of-life wishes can no longer be ascertained. GPs do not act as isolated decision-makers and they believe they act in the best interests of the patient. Advance care planning could help to inform GPs about patients' wishes prior to their loss of competence.

## Background

Studies in several European countries have consistently reported that a number of patients die following end-of-life decisions which may, or are intended to, shorten life. The use of drugs by physicians with the intention of ending a patient's life without his or her explicit request is a practice that has evoked considerable political, ethical and public debate [1-3]. Although legally prohibited all over the world, this practice seems to take place everywhere in modern healthcare, albeit with differences between countries. Incidence estimates within Europe range between 0.06% and 1.50% of all deaths with the highest prevalence being reported in Flanders, Belgium [4-6].

However, despite extensive debate about this practice little is known about its conduct. In Belgium, for instance, it is performed relatively more often among those younger than 80, and those who were judged cognitively incompetent [7-10], and occurs in the home and care home setting under the care of the general practitioner (GP) in about half of all cases in 2001 [9,11,12].

In order to get a comprehensive picture of the last phase of life in these cases, it is indispensable to gain additional insight into the clinical characteristics of the patients, the decision-making process and the performance of the practice. Setting-specific information is also valuable because GP-patient relationships formed over a long period can differ notably from specialist-patient relationships which are often short-term and take place in acute circumstances [13].

This study will focus on the use of life-ending drugs by GPs without explicit patient request, to gain insights into how, why and for which patients GPs decide to end a life in this manner.

Our research questions are:

1. What are the socio-demographical and clinical characteristics of patients dying following the use of life-ending drugs without explicit patient request in general practice in Belgium?

2. Was the decision discussed with patients, family and/or other professional caregivers?

3. Were other end-of-life decisions made and did they precede, follow or take place jointly with the decision to use life-ending drugs?

4. How was the practice performed?

## Methods

### Study design, setting and participants

In 2005 and 2006, a large-scale mortality follow-back study was conducted to monitor end-of-life care and decision-making in Belgium using the Sentinel Network of General Practitioners (SENTI-MELC) study [14]. It involved a quantitative registration study of deaths in the practices of GPs within the Belgian Sentinel Network which, since it was founded in 1979, has proved to be a reliable surveillance system for a wide variety of health-related epidemiological data [14-18] and which is representative of all Belgian GPs in terms of age, sex and region [18,19]. The study resulted in a robust representative sample of non-sudden deaths (n = 1690) [10] not restricted to a specific setting, age group or disease. The study protocol and the first set of results are published elsewhere [10,14,20-22].

During this registration study, we identified deceased patients meeting the following inclusion criteria:

- aged one year or older at time of death

- death did not occur "suddenly or totally unexpectedly" as judged by the GP

- death occurred at home or in a care home

Based on these criteria 225 such patients were identified and a large interview study involving them was performed.

For the current study, patients were included if the GP registered that, in addition:

- death followed the use of 'a drug prescribed, supplied or administered by the GP or a colleague physician with the explicit intention of hastening the end of life'

- the decision concerning this act was made without an explicit request from the patient.

**Figure 1:**
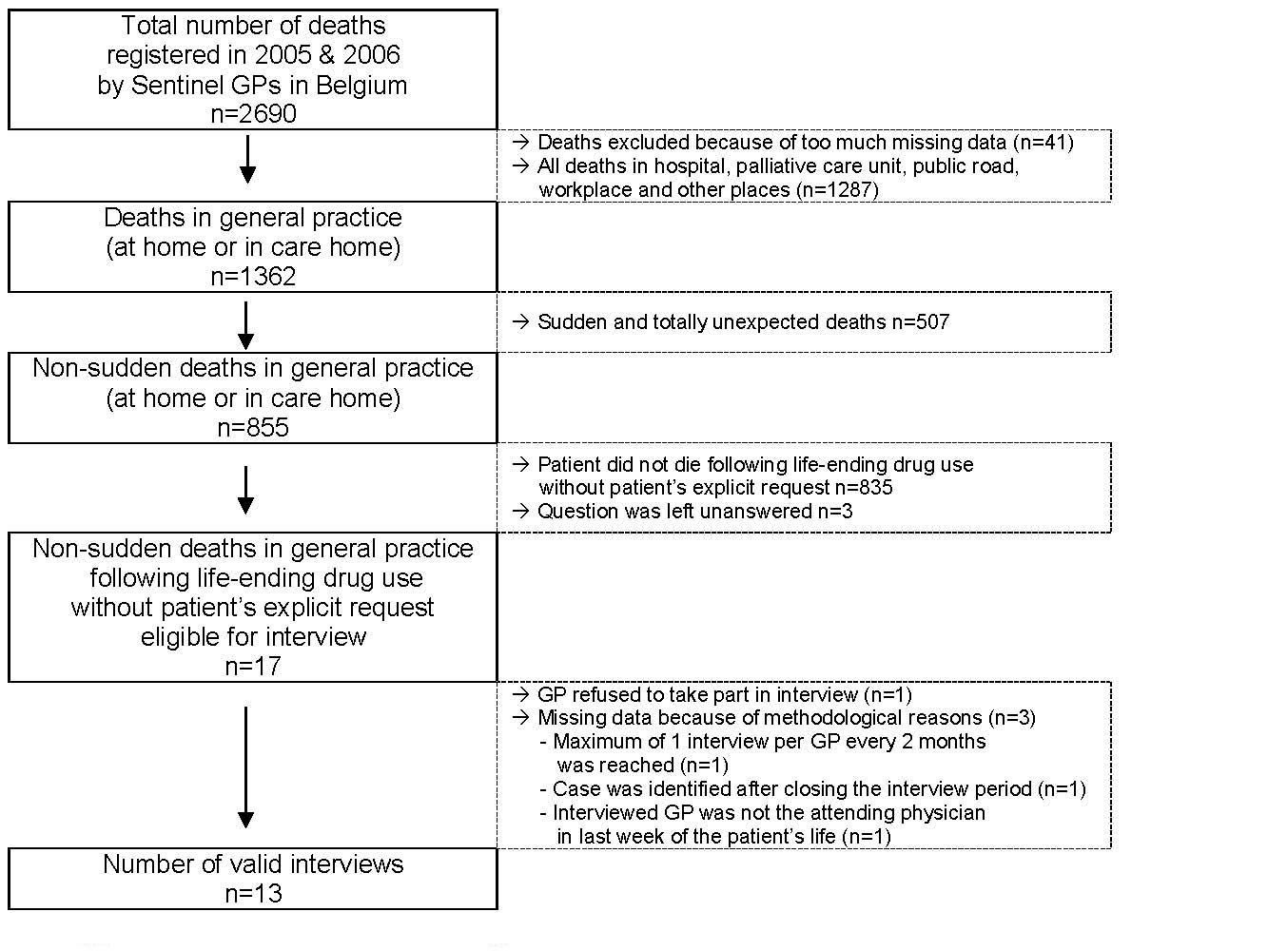
**Process of interview inclusion**.

For 17 (1.3%) out of 1362 patients who died at home or in a care home in Belgium (Figure 1), life-ending drugs without explicit patient request preceded death (binomial 95% CI, exact method: [0.7-2.0]), which is 2.0% of all patients who died in these settings non-suddenly (binomial 95% CI, exact method: [1.2-3.2]). In four cases which met the criteria for inclusion interviews did not take place; in only one of these was the GP unwilling to participate. In total thirteen interviews were conducted.

### Measurements

The interview with each GP was face-to-face and semi-structured and included both closed-ended and open-ended questions. Answers to open-ended questions were written down verbatim. For all questions, there was room to note additional information given by the GP.

Interview questions were based largely on existing questionnaires [1,23-28] (see Table 1 for all interview topics). Information about each patient's socio-demographics such as age at death, sex, level of education, and place of death was retrieved from the SENTI-MELC registration study.

**Table 1:** Interview topics assessed in this study

Questions on the patient's **clinical and care characteristics **during the last phase of life, assessing:
patient's main diagnosis [28]

other diseases for which the patient received treatment during the last three months of life [25-27]

patient's level of consciousness during the last week of life (not unconscious; unconscious one or more hours before death; unconscious one or more days before death; unconscious during whole week) [25-27]

time before death patient had started feeling ill and time before death patient was diagnosed [25-27]

number of GP contacts with the patient or with family regarding the patient during the last 3 months of life

the involvement of informal caregivers and/or clinical specialist in providing care for this patient during the last 3 months of life
symptom burden in the last week of life using an adapted version of the Memorial Symptom Assessment Scale Global Distress Index (MSAS-GDI) [23]

functional status during the last three months of life using the Eastern Cooperative Oncology Group Performance Status Scale (ECOG) [24]

whether or not multidisciplinary palliative care services were involved

whether or not curative, life-prolonging or alternative palliative treatments could be considered that were not applied, and what the reasons were for not applying them [46]

to what extent the patient's suffering was persistent and unbearable and how GPs came to their judgment [46]

to what extent physical and/or psychological suffering was present that could not be alleviated [46]

to what extent the patient's medical situation was without prospect of improvement [46]



Questions on the **process of the decision-making**, assessing [1,3,4,6,25-27,47]:

*The content and timing of the decision-making process:*

whether or not the hastening of death was discussed with the patient (and reason for not discussing)

whether or not the patient was competent to make decisions (and reasons for incompetence)

wishes expressed by the patient concerning the termination of life, prior to the decision-making

involvement in the decision-making of patient's relatives, and other caregivers

time before death the decision was made and

GP's main considerations for doing so

*Whether or not three other types of medical end-of-life decisions were made at the end of the patient's life and their sequence in time in relation to the decision to end life without explicit patient request:*

(1) non-treatment decisions taking into account a possible hastening of death or with the explicit intent to hasten death

(2) intensifying alleviation of pain or other symptoms taking into account or co-intending the hastening of death

(3) using drugs to continuously sedate the patient until death



Questions on the **performance of the practice**, assessing [3,4]:

moment of drug administration and the circumstances surrounding death

drugs used to end life, time between administration of life-ending drugs and coma, and death

persons involved in the drug administration and GP's presence during the period until death

estimated life shortening effect of the drugs

### Procedure

In the SENTI-MELC registration study GPs registered deaths weekly, using a standardised form [14]. Every two months, the registration forms were screened for interview inclusion criteria. The GPs in cases meeting these criteria were contacted by telephone by an independent person to request their participation in a face-to-face interview. The interview took place at a time and place of the GP's choice. Each interview was undertaken by two researchers, one conducting the interview; the other making detailed notes of the interviewees' responses.

Strict procedures were used to preserve patient anonymity and physician confidentiality. Patient names were never identifiable to the interviewers or to other members of the research group: GPs used anonymous codes to refer to their patients in the registration form and the interviewers were given closed envelopes (prepared by the independent telephone operator) to give to the GP before each interview to make sure they referred to the correct patient. After closing the interview study, the GP's identity was permanently deleted from all files. The Ethical Review Board of Brussels University Hospital approved the study protocol.

Several procedures were used to ensure data quality and prevent missing data. If the question to identify patients who died following a life-ending act without explicit request was left unanswered on the registration form, a follow-up letter was sent to the GP. Also, to preclude overburdening the GP, each had no more than one interview per two months and the length of an interview was estimated at a maximum of one hour. In order to prevent recall bias, the interview was arranged as soon as possible after inclusion. Data-entry was done with consistency, range and skip checks, and the data were entered twice.

### Analyses

All closed-ended questions were descriptively analyzed using SPSS 16.0 (SPSS Inc, Chicago, Ill). Results of the study are presented both on an aggregate and on an individual case level. Both answers to open-ended questions and to questions for which additional information was provided by the GPs were encoded into categories by two researchers and/or registered as quotes.

## Results

Table 2 and Additional file 1, Table S1 give an overview of the socio-demographic, care and clinical characteristics of patients dying following life-ending drug use without their explicit request. Of all thirteen patients three were aged 65 or younger at the time of death, eight died at home and five in the care home. Six out of the thirteen patients had been diagnosed with cancer and eleven had suffered from at least one comorbidity within the last three months of life. In the last week of life, all patients were completely bedridden and incapable of self-care, all but one were unconscious or in a coma for one or more hours or days before death, and all experienced symptoms. In general, physical symptoms were reported more often than psychological ones. The most frequently reported physical symptoms were: lack of energy, lack of appetite, feeling drowsy, and pain. The psychological symptoms most frequently reported were feeling nervous and feeling sad. For all but one patient (case n°12) the symptoms also caused serious distress.

**Table 2:** Life-ending drug use in general practice without patient's explicit request - aggregated (n = 13)

Sociodemographic characteristics *		n
Age at death	1-64 years	3
	65-79 years	6
	≥ 80 years	4

Sex	Male	8
	Female	5

Educational level*	Elementary or lower	2
	Lower secondary	6
	Higher secondary or more	4

Community of Belgium	Dutch community	6
	French community	7

Fixed partner at time of death	Yes	6
	No	7

Place of death	Home	8
	Care home	5

**Symptom burden in the last week of life **(MSAS-GDI) †		

Physical symptoms	Lack of energy	12 (6)
	Pain	9 (5)
	Dry mouth	8 (5)
	Difficulty breathing	8 (4)
	Feeling drowsy	10 (3)
	Constipation	7 (2)
	Lack of appetite	11 (1)
Psychological symptoms	Feeling sad	7 (5)
	Feeling nervous	9 (4)
	Worrying	6 (4)
	Feeling irritable	5 (3)

**End-of-life care provision**		

Patient-GP encounters	In last week of life (range)	1-15
	
	In last 3 months of life (range)	6-42

Clinical specialist involved in care in last 3 months of life	None	4
	Sometimes or often	9


Informal care over last 3 months of life	None	1
	Sometimes or often	12

Treatment goal over last 3 months of life	Comfort/palliation	5
	Transition to comfort/palliation	8

Consideration of curative or life-prolonging treatments by GP ‡	Not possible anymore	7
	Still possible but not applied	6
	
Reasons why not applied §	Physician deemed chance for improvement too small	4
	Physician wanted to end further suffering	4
	Patient refused treatment (verbally or non-verbally)	2
	Proxies wanted to end further suffering	1
	Proxies were psychologically and physically exhausted	1

Consideration of alternative palliative treatments by GP ‡	Not possible anymore	10
	Still possible but not applied	3
	
Reasons why not applied §	Physician did not want to prolong patient's life	1
	Physician wanted to end further suffering	2
	Patient refused treatment	1

Multidisciplinary palliative home care team involved in last three months of life	Yes	4
	No	9

* Missing values for level of education n = 1;

† Symptoms that were present during the last week before death, despite possible treatment. Between brackets: symptoms that distressed the patient, if present. Distress levels were measured for all but one case (patient comatose during last week) using the MSAS-GDI. Psychological symptoms were considered to have caused distress if patient did appear to feel this way "frequently" or "almost constantly". Physical symptoms were considered to have caused distress if symptom distressed the patient "quite a bit" or "very much"

‡ At the time of the decision making

§Multiple answers were possible;

For six patients, GPs judged curative or life-prolonging treatments to be available which were not applied for reasons such as affording little chance of improvement or risking additional suffering. In three cases it was considered that palliative treatment options were available but they were not applied because the patient refused further treatment or the physician judged it preferable not to prolong treatment or the life of the patient. Multidisciplinary palliative home care was involved in four cases.

At the time of decision-making, the GP judged the medical situation of all thirteen patients as without any prospect of improvement (Additional file 2, Table S2). Nine were considered to suffer persistently and unbearably to a high or very high degree. All patients suffered physically and/or psychologically to some degree, in ways which could not be alleviated otherwise though one GP deemed the patient's suffering not persistent and unbearable (case n°13). According to this GP *'the suffering was kept under control by medication'*; though any attempts at improving the patient's medical situation had been futile and ending life was '*clearly the best for him' *(qualitative additional information). In all cases GPs based their judgments on observation and compassion; and in ten cases also after conferring with patients themselves (before they lost competence), their loved ones, or with other professional caregivers (not shown in table).

### Decision-making process

**Figure 2:**
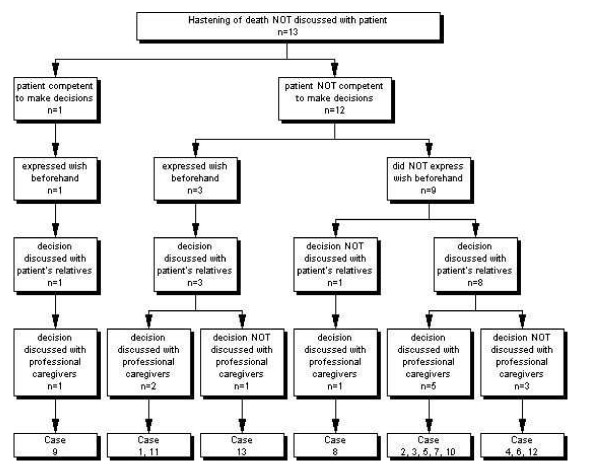
**The discussion of the decision**.

All but one patient had lost the capacity to assess their situation and to make an informed decision about it (Figure 2). Reasons for incompetence cited were: the patient could no longer communicate, or was severely demented, unconscious, mentally disabled or considered too young (not shown in figure). One patient was considered competent but not able to express himself well (case n°9) and had earlier expressed a wish not to suffer anymore although this wish was not an explicit request to hasten death. In this case the medical situation was judged futile to the extent that, in the GP's view, the decision was in the patient's best interest. The GP made the decision in collaboration with a colleague physician and after several discussions with the patient's children.

Nine patients were not competent to make decisions and had not expressed an advance wish about the termination of life. Within this group, the decision to end life was always discussed with either the relatives or a professional caregiver, and mostly with both. In one case no relatives were involved because the patient no longer had family.

In three cases the GP indicated that a wish had been expressed on various occasions while the patient was still competent which, although according to the GP not explicit, bore upon life-ending e.g. *'I do not want to suffer at the end of life*' (qualitative additional information).

Nurses were involved in the decision-making in seven cases for the purpose of exchange of information, joint decision-making, support, or the arrangement of practicalities. Colleague physicians were involved in three cases when they were asked for information, advice, support, or to make the decision jointly. A frequent reason for lack of discussion with a colleague physician was that there was no need or that the situation was clear (not shown in figure).

In seven cases the GP felt influenced by the patient's relatives when making the decision: in five the family was supportive: *'we were on the same wavelength', 'it counts that the family indicates it is taking too long', 'they asked me: can't you do anything?'*. In two cases the GP indicated that the family was initially not ready to consider such a decision, until they were confronted with the increasingly unbearable pain and suffering of their relative (qualitative additional information).

Additional file 3, Table S3 displays other end-of-life decisions made in each case in a chronological manner. In all cases the decision to end life without explicit patient request was preceded by or made jointly with the decision to intensify symptom alleviation. Decisions to withhold or withdraw treatment were also made for ten patients, often at different times. The following treatments were decided to be withheld or withdrawn: the administration of medication (n = 8); artificial hydration or nutrition (n = 7); chemotherapy or radiotherapy (n = 3); reanimation (n = 2); a blood transfusion (n = 1); the performance of an operation (n = 1).

In seven cases the GP decided to sedate the patient continuously and deeply until death, and in five of these this decision was made at the same time as the use of life-ending drugs. While for most patients the decision to end life was made within the last weeks or days, two GPs - in collaboration with the patient's relatives - made it more than one month before death. For one patient (case n°12), the relevant drugs (morphine and dormicum) were made available seven months prior to administration in the event that the brain tumor made suffering unbearable without possible alleviation which was the case in the end. For one other patient (case n°9), the drugs were available for several weeks before death and kept in the patient's house, in case the relatives agreed upon on ending the patient's life; the GP told them: *'it is you who has to decide' *(qualitative additional information).

### Characteristics of the performance of the practice

None of the patients was competent at the time of the administration of the drugs (Table 3). The hastening of death involved the administration of opioids in all but one case; seven patients received no other drug but opioids, and opioids were combined with a benzodiazepine in five other cases. A barbiturate induced death for one patient (case n°10). Neuromuscular relaxants were not used at all.

**Table 3:** Life-ending drug use without patient's explicit request: performance of the practice (n = 13)

			n
Patient was competent at time of drug administration			0

Drugs used to end life		Opioids only	7
		Opioids in combination with a benzodiazepine	5
		Barbiturate	1
	
	In case opioids were used to end life (n = 12)	Use of opioids already administered previous to	9
		life-ending practice to alleviate pain or other symptoms	
	
	Time between administration of (first) life-ending drug and coma	Patient was already in a coma at time of administration	6
		Patient never lapsed into a complete coma	1
		(awoke now and then)	
		15 minutes	1
		2 hours	1
		8 hours	1
		1 day	1
		2 days	2
	
	Time between administration of (first) life-ending drug and death	instantly	1
		20 minutes	1
		90 minutes	2
		3 hours	1
		4 hours	1
		12 hours	1
		13 hours	1
		1-2 days	3
		2-3 days	2

Persons involved in administration	Person who administered the (last) life-ending drug	GP	7
		Nurse	6
	
	GP's presence during the administration of life-ending drugs until the time of death	Continuously	2
		With short interruptions	3
		Not present, but on call	6
		Not present	2
	
	Other persons present during the administration of life-ending drug	Professional caregivers & patient's relatives	2
		Professional caregivers only	3
		Patient's relatives only	7
		No other persons present	1

GP's estimation of life-shortening effect of administration of life-ending drugs		< 1 day	2
		1-7 days	8
		1-4 weeks	2
		> 6 months	1

In three quarters of cases where the GP indicated opioids were used to end life, they had already been administered previously to alleviate pain or other symptoms. In six cases, patients were already in a coma at the time the first drug was administered and most others lapsed into a coma within the following hours. All patients died within three days of the first drug being administered. Three GPs reported having technical problems during the administration: one reported the occurrence of unexpected spasms (case n°7), one expected death to occur more rapidly (case n°3), and one expected it to occur more slowly (case n°6) (not shown in table).

In seven cases the final drug was given by the GP and in six by a nurse (case n°3/4/5/6/7/8). For all patients for whom death occurred in a care home a nurse administered the drug without presence of the GP although the GP was on call in four out of five of these cases (not shown in table). Relatives of the patient were present during the administering of the drug in nine cases. The estimated life-shortening effect was for all but one patient less than one month. This one patient (case n°10) had lost all brain function several months before death and had been held in a coma ever since (qualitative additional information).

All GPs said that the instigation of life-ending had improved their patients' end-of-life quality to some or to a considerable extent.

## Discussion

In this interview study, we examined the practice of life-ending drug use without explicit patient request in thirteen cases in general practices in Belgium. The GPs involved indicated that at the time of making the decision there was no realistic prospect of improvement in the condition of any of these patients, most of whom who were suffering persistent and unbearable pain. Almost all patients had lost the capacity to assess their situation and to make decisions. In all cases the life-ending decision was discussed with other caregivers and/or those close to the patient and had preceded, accompanied or was followed by at least one other end-of-life decision. All but one patient received opioids with the explicit intention of hastening the end of life. GPs believed without exception that the patient's end-of-life quality had been improved considerably by their actions.

This is the first study to report on the practice of life-ending drug use without explicit patient request and to provide detailed information on an individual level on clinical characteristics during the last phase of life, on the decision-making process and on the performance of the practice. One major strength is that the thirteen cases were selected from a large two-year registration study, representative of all non-sudden deaths in the country [10], and gathered via a nationwide Sentinel Network representative of all GPs in Belgium. As such, the number of patients dying non-suddenly following life-ending drug use without explicit request in general practice (2.0%) was very similar to estimated incidence figures described in other studies in Belgium in 2001 (2.3%) [12]. Furthermore, the collected data are considered to be of high quality because the cooperation of the GPs in the network is optimal, because all interviews were conducted face to face by two researchers and as soon as possible after inclusion, and because quality control measures were used in both the registration and the interview study.

Finally, since the incidence of life-ending drug use has been shown to be relatively high compared with other countries [4-6] this study of Belgium is of particular interest.

However, because of the small sample of cases the results have to be interpreted cautiously. Notwithstanding that 13 interviews out of a possible 17 were conducted, the four additional interviews could probably have provided even more insight into the nature of this delicate practice. Also, due to the retrospective design of the study a possible recall bias could not be excluded entirely, or some decisions might have been interpreted differently a posteriori. Finally, our findings remain limited to the experiences of GPs. Views of patients, their family, and other caregivers were not studied.

The current study shows that patients who die following the practice of life-ending drug use without explicit request are those who suffer from incurable lingering diseases and whose quality of life dwindles drastically in the last phase. Although some patients were still fully active and ambulatory during the third month before death, they were all completely bedridden and incapable of self-care in the last week of life. Their medical situation within the final days was characterized mainly by unbearable and persistent suffering, characterized by physical as well as psychological symptom burden or by intermittent or permanent unconsciousness. That being in a coma is seen as a kind of suffering might seem inconsistent, but this might be explained by a subjective, compassionate interpretation GPs make at this very end of their long-lasting relationship with the patient. As the GPs believed without exception that their patient's end-of-life quality had been improved considerably by taking this step, it appears that they acted out of compassion and chose what they believed to be the least bad option in a medically futile situation. Whether compassion alone may in some cases justify this practice remains subject to intense debate however [29].

Another important finding of the study is that, even though patients did not or were not able to make an explicit life-ending request, GPs were in many cases unaware of their patient's wishes. This is remarkable since 95% of the population has a regular GP in Belgium [30] and have often built up a long-lasting relationship over the course of many years [31,32]. An explanation may be found in previous research that indicated that GPs experience uncertainty about initiating end-of-life discussions with their patients [33-35] which could mean that they wait for the moment at which decision-making becomes relevant, by which time patients may no longer have the capacity to express their wishes themselves. Therefore, advance care planning, i.e. communication with patients to explore their wishes in the case that they become unable to participate in decision-making [36], remains a matter of great concern in current general practice as GPs are in a key position to initiate and facilitate such discussions [37-40].

Although consultation rarely takes place with patients themselves, it does occur frequently with others: GPs do not appear to act surreptitiously or as isolated decision-makers but to involve both other professional caregivers and the patient's close circle in the decision-making process. This might suggest that GPs have a need for the exchanging of information, for consultation and advice, and for making these decisions jointly with others. However, it should be noted that in some cases the GP felt no need to discuss the decision with other professional caregivers. Furthermore, it is remarkable that multidisciplinary palliative home care teams are not consulted more often within the last months of these patients' lives, even though in Belgium they are available to all GPs in the country. The question however remains as to whether the involvement of such teams would have led to a different end-of-life decision.

Several of our findings further suggest that this end-of-life practice is quite complex and that for the GPs involved it resembles the process of intensified symptom alleviation with a possible life-shortening effect or the process of continuous deep sedation until death rather than a separate and lethal act such as euthanasia. Firstly, such decisions (symptom alleviation/sedation) were made in all cases in addition to the explicit decision to end the patient's life, and often at the same time. Therefore it seems that these medications were often given with a dual purpose: alleviating symptoms and hastening the end of life. Secondly, death did not occur immediately after the administration of the drugs. In a substantial number of cases several hours or days passed by before the patient died. This is in contradiction to what could be expected where a practice such as euthanasia was intended. Thirdly, even though there is strong evidence that the lethal potential of opioids and sedatives is doubtful and they therefore considered unsuitable agents where the hastening of death is explicitly intended [41-43], opioids, whether or not in combination with a benzodiazepine, were the predominant drug used. In addition, opioids were often already being administered to alleviate pain and symptoms prior to the life-ending action. The use of a neuromuscular relaxant which is noted in literature as an efficient euthanaticum with immediate life-shortening effect [44] was not administered in any of the reported cases. Other studies confirm that opioids are commonly in this practice [6,45].

## Conclusion

In conclusion, this study shows that the practice of life-ending drug use without explicit patient request in general practice often seems to be an act of compassion to end the unbearable suffering of patients who can no longer decide for themselves. Our study provides various indications that the line between different end-of-life decisions is not always easy to define as the clinical context of this practice leans towards the process of intensified symptom alleviation or of continuous deep sedation until death. Although GPs are often not aware of their patient's end-of-life wishes, they do not act as isolated decision-makers and involve other professional caregivers and the patient's close circle in the decision. Advance care planning could inform GPs about their patient's wishes before the patient becomes incompetent.

## Competing interests

The authors declare that they have no competing interests.

## Authors' contributions

KM had full access to all the data in the study and takes responsibility for the integrity of the data and the accuracy of the data analysis, and drafted and revised the manuscript. LVDB contributed to the design, coordination and funding of the study, the analysis of the data and to the drafting and revisions of the manuscript. NB and JB participated in the design and coordination of the study, and contributed to the interpretation of the data, and critically revised the manuscript. ME contributed to the interpretation of the data, and critically revised the manuscript. LD contributed to the design, coordination and funding of the study, contributed to the interpretation of the data, and critically revised the manuscript. All authors read and approved the final manuscript.

## Pre-publication history

The pre-publication history for this paper can be accessed here:

http://www.biomedcentral.com/1471-2458/10/186/prepub

## Supplementary Material

Additional file 1 **Table S1**. Life-ending drug use in general practice without patient's explicit request: patients' clinical characteristics during the last phase of life - case level (n = 13). Click here for file

Additional file 2 **Table S2**. Life-ending drug use in general practice without patient's explicit request: patient's suffering at the time of the decision-making (n = 13). Click here for file

Additional file 3 **Table S3**. Life-ending drug use without patient's explicit request and the process of decision-making: timing and involvement of other end-of-life decisions (n = 13). Click here for file

## References

[B1] Deliens L Mortier F Bilsen J Cosyns M Vander Stichele R Vanoverloop J End-of-life decisions in medical practice in Flanders, Belgium: a nationwide survey Lancet 2000 356 1806 1811 10.1016/S0140-6736(00)03233-5 11117913

[B2] Onwuteaka-Philipsen BD van der Heide A Koper D Keij-Deerenberg I Rietjens JA Rurup ML Euthanasia and other end-of-life decisions in the Netherlands in 1995, and 2001 Lancet 1990 362 395 399 10.1016/S0140-6736(03)14029-9 12907015

[B3] van der Maas PJ van der Wal G Haverkate I de Graaff CL Kester JG Onwuteaka-Philipsen BD Euthanasia, physician-assisted suicide, and other medical practices involving the end of life in the Netherlands, 1990-1995 N Engl J Med 1996 335 1699 1705 10.1056/NEJM199611283352227 8929370

[B4] van der Heide A Deliens L Faisst K Nilstun T Norup M Paci E End-of-life decision-making in six European countries: descriptive study Lancet 2003 362 345 350 10.1016/S0140-6736(03)14019-6 12907005

[B5] Seale C National survey of end-of-life decisions made by UK medical practitioners Palliat Med 2006 20 3 10 10.1191/0269216306pm1094oa 16482752

[B6] van der Heide A Onwuteaka-Philipsen BD Rurup ML Buiting HM van Delden JJ Hanssen-de Wolf JE End-of-life practices in the Netherlands under the Euthanasia Act N Engl J Med 2007 356 1957 1965 10.1056/NEJMsa071143 17494928

[B7] Van den Block L Bilsen J Deschepper R Van Der Kelen G Bernheim JL Deliens L End-of-life decisions among cancer patients compared with noncancer patients in Flanders, Belgium J Clin Oncol 2006 24 2842 2848 10.1200/JCO.2005.03.7531 16782923

[B8] De Gendt C Bilsen J Mortier F Vander Stichele R Deliens L End-of-life decision-making and terminal sedation among very old patients Gerontology 2008 55 99 105 10.1159/000163445 18843178

[B9] Rietjens JA Bilsen J Fischer S van der Heide A van der Maas PJ Miccinessi G Using drugs to end life without an explicit request of the patient Death Stud 2007 31 205 221 10.1080/07481180601152443 17330359

[B10] Van den Block L Deschepper R Bilsen J Bossuyt N Van Casteren V Deliens L Euthanasia and other end-of-life decisions: a mortality follow-back study in Belgium BMC Public Health 2009 9 79 10.1186/1471-2458-9-79 19272153 PMC2660906

[B11] Bilsen J Vander Stichele R Mortier F Bernheim J Deliens L The incidence and characteristics of end-of-life decisions by GPs in Belgium Fam Pract 2004 21 282 289 10.1093/fampra/cmh312 15128690

[B12] Cohen J Bilsen J Fischer S Lofmark R Norup M van der HA End-of-life decision-making in Belgium, Denmark, Sweden and Switzerland: does place of death make a difference? J Epidemiol Community Health 2007 61 1062 1068 10.1136/jech.2006.056341 18000128 PMC2465676

[B13] Van den Block L Deschepper R Bilsen J Van Casteren V Deliens L Transitions between care settings at the end of life in Belgium JAMA 2007 298 1638 1639 10.1001/jama.298.14.1638 17925515

[B14] Van den Block L Van Casteren V Deschepper R Bossuyt N Drieskens K Bauwens S Nationwide monitoring of end-of-life care via the Sentinel Network of General Practitioners in Belgium: the research protocol of the SENTI-MELC study BMC Palliat Care 2007 6 6 10.1186/1472-684X-6-6 17922893 PMC2222051

[B15] Fleming DM Schellevis FG Van Casteren V The prevalence of known diabetes in eight European countries Eur J Public Health 2004 14 10 14 10.1093/eurpub/14.1.10 15080383

[B16] Devroey D Van Casteren V Buntinx F Registration of stroke through the Belgian sentinel network and factors influencing stroke mortality Cerebrovasc Dis 2003 16 272 279 10.1159/000071127 12865616

[B17] Stroobant A Van Casteren V Thiers G Eylenbosch WJ, Noah D Surveillance systems from primary-care data: surveillance through a network of sentinal general practitioners Surveillance in Health and Disease 1988 Oxford: Oxford University Press 62 74

[B18] Lobet MP Stroobant A Mertens R Van Casteren V Walckiers D Masuy-Stroobant G Tool for validation of the network of sentinel general practitioners in the Belgian health care system Int J Epidemiol 1987 16 612 618 10.1093/ije/16.4.612 3440673

[B19] Boffin N Bossuyt N Van Casteren V Current characteristics and evolution of the Sentinel General Practitioners: data gathered in 2005 [Huidige kenmerken en evolutie van de peilartsen en hun praktijk. Gegevens verzameld in 2005] IPH/EPI REPORTS N° 2007 - 013 2007 Scientific Institute of Public Health Belgium; Unit of Epidemiology http://www.iph.fgov.be/epidemio/epien/index10.htm

[B20] Van den Block L End-of-life care and medical decision-making in the last phase of life 2008 Brussels, VUB Press

[B21] Van den Block L Deschepper R Bilsen J Van Casteren V Deliens L Transitions between care settings at the end of life in belgium JAMA 2007 298 1638 1639 10.1001/jama.298.14.1638 17925515

[B22] Block L Van den Deschepper R Drieskens K Bauwens S Bilsen J Bossuyt N Hospitalisations at the end of life: using a sentinel surveillance network to study hospital use and associated patient, disease and healthcare factors BMC Health Serv Res 2007 7 69 10.1186/1472-6963-7-69 17488520 PMC1885255

[B23] Hickman SE Tilden VP Tolle SW Family reports of dying patients' distress: the adaptation of a research tool to assess global symptom distress in the last week of life J Pain Symptom Manage 2001 22 565 574 10.1016/S0885-3924(01)00299-8 11516598

[B24] Oken MM Creech RH Tormey DC Horton J Davis TE McFadden ET Toxicity and response criteria of the Eastern Cooperative Oncology Group Am J Clin Oncol 1982 5 649 655 10.1097/00000421-198212000-00014 7165009

[B25] A controlled trial to improve care for seriously ill hospitalized patients. The study to understand prognoses and preferences for outcomes and risks of treatments (SUPPORT). The SUPPORT Principal Investigators JAMA 1995 274 1591 1598 10.1001/jama.274.20.1591 7474243

[B26] Klinkenberg M The last phase of life of older people: health, preferences and care: a proxy report study 2003 Amsterdam, The Netherlands: PhD thesis, EMGO Institute, VU Amsterdam

[B27] Deeg D Beekman A Kriegsman D Westendorp-de Serière M Autonomy and well-being in the aging population II: Report from the Longitudinal Aging Study Amsterdam 1992-1996 1998 Amsterdam: VU University Press

[B28] van der Wal G van der Heide A Onwuteaka-Philipsen BD van der Maas PJ Medical decision-making at the end of life: practice in The Netherlands and the evaluation procedure of euthanasia [Medische besluitvorming aan het einde van het leven: de praktijk en de toetsingsprocedure euthanasie] 2003 Utrecht, The Netherlands: De Tijdstroom Uitgeverij

[B29] Materstvedt LJ Palliative care on the 'slippery slope' towards euthanasia? Palliat Med 2003 17 387 392 10.1191/0269216303pm796oa 12882253

[B30] Bayingana K Demarest S Gisle L Hesse E Miermans PJ Tafforeau J Health survey interview, Belgium 2004. Depotn°: D/2006/2505/4, IPH/EPI REPORTS N° 2006 - 035 2006 Scientific Institute of Public Health Belgium, Department of Epidemiology

[B31] Munday D Dale J Murray S Choice and place of death: individual preferences, uncertainty, and the availability of care J R Soc Med 2007 100 211 215 10.1258/jrsm.100.5.211 17470927 PMC1861422

[B32] Michiels E Deschepper R Van Der Kelen G Bernheim J Mortier F Vander Stichele R The role of general practitioners in continuity of care at the end of life: a qualitative study of terminally ill patients and their next of kin Palliat Med 2007 00 1 7 10.1177/0269216307078503 17901100

[B33] Tierney WM Dexter PR Gramelspacher GP Perkins AJ Zhou XH Wolinsky FD The effect of discussions about advance directives on patients' satisfaction with primary care J Gen Intern Med 2001 16 32 40 10.1111/j.1525-1497.2001.00215.x 11251748 PMC1495157

[B34] Deschepper R Vander Stichele R Bernheim JL De Keyser E Van Der Kelen G Mortier F Communication on end-of-life decisions with patients wishing to die at home: the making of a guideline for GPs in Flanders, Belgium Br J Gen Pract 2006 56 14 19 16438810 PMC1828069

[B35] Brown M Participating in end of life decisions. The role of general practitioners Aust Fam Physician 2002 31 60 62 11840892

[B36] Teno JM Nelson HL Lynn J Advance care planning. Priorities for ethical and empirical research Hastings Cent Rep 1994 24 S32 S36 10.2307/3563482 7860278

[B37] Emanuel LL Danis M Pearlman RA Singer PA Advance care planning as a process: structuring the discussions in practice J Am Geriatr Soc 1995 43 440 446 7706637 10.1111/j.1532-5415.1995.tb05821.x

[B38] Aitken PVJr Incorporating advance care planning into family practice [see comment] Am Fam Physician 1999 59 605 620 10029787

[B39] Gallagher R An approach to advance care planning in the office Can Fam Physician 2006 52 459 464 16639971 PMC1481678

[B40] Cartwright CM Parker MH Advance care planning and end of life decision making Aust Fam Physician 2004 33 815 819 15532156

[B41] Sykes N Thorns A The use of opioids and sedatives at the end of life Lancet Oncol 2003 4 312 318 10.1016/S1470-2045(03)01079-9 12732169

[B42] Thorns A Sykes N Opioid use in last week of life and implications for end-of-life decision-making Lancet 2000 356 398 399 10.1016/S0140-6736(00)02534-4 10972375

[B43] Sykes N Thorns A Sedative use in the last week of life and the implications for end-of-life decision making Arch Intern Med 2003 163 341 344 10.1001/archinte.163.3.341 12578515

[B44] Royal Dutch Society for the Advancement of Pharmacy (KNMP). Utilization and Preparation of Euthanasia Drugs [In Dutch] 1998 The Hague, The Netherlands

[B45] Rurup ML Borgsteede SD van der Heide A van der Maas PJ Onwuteaka-Philipsen BD Trends in the use of opioids at the end of life and the expected effects on hastening death J Pain Symptom Manage 2009 37 144 155 10.1016/j.jpainsymman.2008.02.010 18692359

[B46] Belgisch Staatsblad 22 juni 2002 [Belgian official collection of the laws June 22 2002]. Wet betreffende euthanasie 28 mei 2002 [Law concerning euthanasia May 28, 2002] (in Dutch). 2002009590 2002

[B47] Mitchell K Owens RG National survey of medical decisions at end of life made by New Zealand general practitioners BMJ 2003 327 202 203 10.1136/bmj.327.7408.202 12881263 PMC166121

