# Supplementary material for: Physician reports of medication use with explicit intention of hastening the end of life in the absence of explicit patient request in general practice in Belgium
Source: BMC Public Health. 2010 Apr 9;10:186. doi: 10.1186/1471-2458-10-186 (PMC2867997; doi:10.1186/1471-2458-10-186)
Supplement: Additional file 1 — Table S1. Life-ending drug use in general practice without patient's explicit request: patients' clinical characteristics during the last phase of life - case level (n = 13). [file 1471-2458-10-186-S1.DOC]

**Table S1. Life-ending drug use in general practice without patient’s explicit request: patients’ clinical characteristics during the last phase of life – case level (n=13)**

| **Case**  **n°** | **main**  **diagnosis** | **time before death patient started**  **feeling ill** | | **performance status**  **in 2^nd^ and 3^rd^ month before death** ‡ | **performance**  **status**  **during last week** | **number of symptoms that caused distress during last week §** | **symptom that caused**  **most distress**  **during last week** | **unconscious or**  **in a coma**  **during last week** |
| --- | --- | --- | --- | --- | --- | --- | --- | --- |
|  |  |  | |  |  |  |  |  |
| **1** | COPD * | > | 6 months | 4 | 4 | 8 | difficulty breathing | last hours |
| **2** | cancer † |  | 5 months | 1 | 4 | 7 | lack of energy | last hours |
| **3** | dementia * | > | 6 months | 4 | 4 | 1 | dry mouth | never |
| **4** | cancer * |  | 3 months | 3 | 4 | 4 | feeling irritable | last day(s) |
| **5** | dementia * | > | 6 months | 1 | 4 | 5 | pain | last hours |
| **6** | pneumonia * |  | 2 weeks | 0 | 4 | 1 | difficulty breathing | last day(s) |
| **7** | cardiovascular * |  | 4 months | 3 | 4 | 6 | pain | last day(s) |
| **8** | CVA * |  | 4 weeks | 2 | 4 | 1 | dry mouth | last hours |
| **9** | cancer * † | > | 6 months | 1 | 4 | 4 | worrying | last hours |
| **10** | encephalitis * |  | 5 months | 4 | 4 | not applicable | not applicable | entire week |
| **11** | cancer * † | > | 6 months | 2 | 4 | 3 | lack of energy | last hours |
| **12** | cancer | > | 6 months | 4 | 4 | 0 | not applicable | last day(s) |
| **13** | cancer * † |  | 5 months | 1 | 4 | 2 | lack of energy | last hours |
|  |  |  | |  |  |  |  |  |

* With long-lasting comorbidities within the last three months of life;

† With metastases;

‡ Performance Status Scale (ECOG) 0-Fully active; 1-Ambulatory, capable of work of a light nature; 2-Capable of self-care but not work; 3-In bed ≥50% of the time, capable of only limited self-care; 4-Completely bedridden, incapable of self-care;

§ Despite possible treatment. Distress levels were measured using the Memorial Symptom Assessment Scale-GDI (measuring 11 possible symptoms) except for patients who were in a state of unconsciousness during the entire last week of life (n=1). Psychological symptoms were considered to have caused distress if patient did appear to feel this way “frequently” or “almost constantly”. Physical symptoms were considered to have caused distress if symptom distressed the patient “quite a bit” or “very much”
