# Supplementary material for: Physician reports of medication use with explicit intention of hastening the end of life in the absence of explicit patient request in general practice in Belgium
Source: BMC Public Health. 2010 Apr 9;10:186. doi: 10.1186/1471-2458-10-186 (PMC2867997; doi:10.1186/1471-2458-10-186)
Supplement: Additional file 2 — Table S2. Life-ending drug use in general practice without patient's explicit request: patient's suffering at the time of the decision-making (n = 13). [file 1471-2458-10-186-S2.DOC]

**Table S2. Life-ending drug use in general practice without patient’s explicit request: patient’s suffering at the time of the decision-making (n=13)**

| **Case n°** | **suffering was persistent and unbearable** | **unbearable physical**  **suffering that could not be alleviated** | **unbearable psychological suffering that could not be alleviated** | **medical situation was without prospect of improvement** | **Total number of (+++ or ++)**  **within each case** |
| --- | --- | --- | --- | --- | --- |
| **1** | +++ | +++ | +++ | +++ | **4** |
| **2** | +++ | +++ | ++ | +++ | **4** |
| **3** | +++ | +++ | +++ | +++ | **4** |
| **4** | ++ | +++ | ++ | +++ | **4** |
| **5** | ++ | ++ | ++ | +++ | **4** |
| **6** | +++ | +++ | + | +++ | **3** |
| **7** | +++ | +++ | + | +++ | **3** |
| **8** | ++ | + | +++ | +++ | **3** |
| **9** | ++ | + | + | +++ | **2** |
| **10** | + | + | +++ | ++ | **2** |
| **11** | + | ++ | + | +++ | **2** |
| **12** | + | + | +++ | +++ | **2** |
| **13** | 0 | + | 0 | +++ | **1** |
| **Total number of cases** |  |  |  |  |  |
| **with (+++ or ++)** | **9** | **8** | **8** | **13** |  |

Key to symbols: +++ (to a very high degree); ++ (to a high degree); + (to a lesser degree); 0 (no suffering)
