# Supplementary material for: Physician reports of medication use with explicit intention of hastening the end of life in the absence of explicit patient request in general practice in Belgium
Source: BMC Public Health. 2010 Apr 9;10:186. doi: 10.1186/1471-2458-10-186 (PMC2867997; doi:10.1186/1471-2458-10-186)
Supplement: Additional file 3 — Table S3. Life-ending drug use without patient's explicit request and the process of decision-making: timing and involvement of other end-of-life decisions (n = 13). [file 1471-2458-10-186-S3.DOC]

|  | Time before death that decision to use life-ending drugs without explicit patient request was made | Other end-of-life decision(s) *preceding*  the decision to use life-ending drugs  without explicit patient request | | | |  | Other end-of-life decision(s) *made jointly with*  the decision to use life-ending drugs without explicit patient request | | | | Other end-of-life decision(s) *following*  the decision to use life-ending drugs  without explicit patient request | | | | |  | At least  one other end-of-life decision was made |
| --- | --- | --- | --- | --- | --- | --- | --- | --- | --- | --- | --- | --- | --- | --- | --- | --- | --- |
|  |  |  |  |  |  |  |  |  |  |  |  |  |  |  |  |  |  |
| Case n° |  | withholding / withdrawing treatment † | intensified symptom alleviation ‡ | continuous deep sedation until death | **In**  **total** |  | withholding / withdrawing treatment † | intensified symptom alleviation ‡ | continuous deep sedation until death | **In**  **total** |  | withholding / withdrawing treatment † | intensified symptom alleviation ‡ | continuous deep sedation until death | **In**  **total** |  |  |
| **1** | **≤ 1 day** |  |  |  |  |  |  | + |  | **+** |  |  |  |  |  |  | **+** |
| **6** | **≤ 1 day** | + |  |  | **+** |  |  | + |  | **+** |  |  |  |  |  |  | **+** |
| **13*** | **≤ 1 day** |  | + | + | **+** |  |  |  |  |  |  |  |  |  |  |  | **+** |
| **4** | **2-7 days** | + |  |  | **+** |  |  | + |  | **+** |  |  |  |  |  |  | **+** |
| **2** | **2-7 days** | + |  |  | **+** |  | + | + | + | **+** |  | + |  |  | **+** |  | **+** |
| **3** | **2-7 days** | + |  |  | **+** |  | + | + | + | **+** |  |  |  |  |  |  | **+** |
| **8** | **2-7 days** | + | + |  | **+** |  |  |  |  |  |  |  |  |  |  |  | **+** |
| **10** | **1-4 weeks** |  | + | + | **+** |  |  |  |  |  |  | + |  |  | **+** |  | **+** |
| **7** | **1-4 weeks** | + |  |  | **+** |  |  | + | + | **+** |  | + |  |  | **+** |  | **+** |
| **11** | **1-4 weeks** | + |  |  | **+** |  | + | + | + | **+** |  | + |  |  | **+** |  | **+** |
| **5** | **1-4 weeks** |  |  |  |  |  | + | + |  | **+** |  |  |  |  |  |  | **+** |
| **9** | **1-2 months** |  |  |  |  |  |  | + |  | **+** |  |  |  |  |  |  | **+** |
| **12** | **> 6 months** |  |  |  |  |  |  | + | + | **+** |  |  |  |  |  |  | **+** |
|  | **In total** | n=7 | n=3 | n=2 | **n=9** | n=4 | | n=10 | n=5 | **n=10** | n=4 | | n=0 | n=0 | **n=4** | **n=13** | |

Table S3. Life-ending drug use without patient’s explicit request and the process of decision-making: timing and involvement of other end-of-life decisions (n=13)

* A decision to withhold/withdraw treatment was made but moment of decision-making unknown;

† Taking into account or explicitly intending hastening of death

‡ Taking into account or co-intending hastening of death
